# Drug-Loaded Biocompatible Nanocarriers Embedded in Poloxamer 407 Hydrogels as Therapeutic Formulations

**DOI:** 10.3390/medicines6010007

**Published:** 2018-12-29

**Authors:** Elena Giuliano, Donatella Paolino, Massimo Fresta, Donato Cosco

**Affiliations:** 1 Department of Health Sciences, University “Magna Græcia” of Catanzaro, Campus Universitario “S. Venuta”, Viale S. Venuta, I-88100 Catanzaro, Italy; elena.giuliano@unicz.it (E.G.); fresta@unicz.it (M.F.); 2 Department of Experimental and Clinical Medicine, University “Magna Græcia” of Catanzaro, Campus Universitario “S. Venuta”, Viale S. Venuta, I-88100 Catanzaro, Italy; paolino@unicz.it

**Keywords:** poloxamer 407, colloids, liposomes, niosomes, ethosomes, nanoparticles, controlled drug release

## Abstract

Hydrogels are three-dimensional networks of hydrophilic polymers able to absorb and retain a considerable amount of water or biological fluid while maintaining their structure. Among these, thermo-sensitive hydrogels, characterized by a temperature-dependent sol–gel transition, have been massively used as drug delivery systems for the controlled release of various bioactives. Poloxamer 407 (P407) is an ABA-type triblock copolymer with a center block of hydrophobic polypropylene oxide (PPO) between two hydrophilic polyethyleneoxide (PEO) lateral chains. Due to its unique thermo-reversible gelation properties, P407 has been widely investigated as a temperature-responsive material. The gelation phenomenon of P407 aqueous solutions is reversible and characterized by a sol–gel transition temperature. The nanoencapsulation of drugs within biocompatible delivery systems dispersed in P407 hydrogels is a strategy used to increase the local residence time of various bioactives at the injection site. In this mini-review, the state of the art of the most important mixed systems made up of colloidal carriers localized within a P407 hydrogel will be provided in order to illustrate the possibility of obtaining a controlled release of the entrapped drugs and an increase in their therapeutic efficacy as a function of the biomaterial used.

## 1. Introduction

Nanosystems containing drugs are important formulations used for the clinical treatment of various diseases. They offer important advantages such as specific cell/tissue targeting, prolonged systemic circulation, protection from metabolic modification, improved bioavailability, and increased therapeutic efficiency of entrapped/complexed bioactive compounds [1,2]. They have been successfully employed in therapeutic strategies for a wide range of infectious and non-infectious diseases (cancer, atherosclerosis, diabetes) [3]. The intravenous administration of drugs encapsulated in the nanocarriers promotes their localization in specific tissues as a consequence of the anatomical functions and physiological characteristics of the various body compartments, but is totally unsuitable when a local effect and sustained drug release are necessary [4]. 

Hydrogels are three-dimensional networks made up of hydrophilic polymers capable of absorbing and retaining considerable amounts of water or biological fluids [5,6]. This property is due to the presence of hydrophilic functional residues (e.g. -OH, -CONH_2_, and -SO_3_H) in the polymeric backbone [7,8] and to the crosslinking of the network chains [9]. Chemical interactions (covalent bonds, hydrogen binding, and van der Waals forces) or physical entanglements are involved in the composition of the structure [7,10]. Hydrogels can be classified as a function of various characteristics, such as the source (natural, synthetic, or hybrid), the polymeric composition (homo-, co-, or multipolymer derivatives), the physico-chemical configuration (amorphous, semicrystalline, and crystalline), and responsiveness to physiological environmental stimuli (e.g., pH, ionic strength, and temperature) [9,11].

Due to their peculiar properties, such as biocompatibility, biodegradability, and good tolerability [12], they have been proposed and used as promising biomaterials for a wide range of applications, including tissue engineering and regenerative medicine, the manufacture of contact lenses, diagnostics, and drug delivery systems [13,14,15]. Temperature-responsive hydrogels have stimulated great interest in pharmaceutical applications because they can be applied in situ forming systems. In particular, they are fluids at room temperature, thus favoring ease of injection into tissues, organs, or body cavities, while they gel at body temperature, allowing a localized and sustained release of drugs, decreasing the number of necessary doses and, consequently, reducing the potential drug-related side effects [12]. Various temperature-responsive materials have been used in the making of hydrogels, such as poloxamers, which are ABA-type triblock copolymers made up of a hydrophobic block of polypropylene oxide (PPO) between two hydrophilic polyethyleneoxide (PEO) chains, and are versatile materials used to develop different types of these formulations [16]. Among these, poloxamer 407 (P407) has been widely studied as a potential biomaterial for obtaining thermo-sensitive hydrogels thanks to its solubilizing capacity, low toxicity, drug release characteristics, and compatibility with numerous biomolecules and excipients [17,18].

Despite their numerous advantageous properties, hydrogels have several limitations such as a restricted drug-loading content (especially in the case of hydrophobic compounds), rapid dissolution, and relatively rapid drug release [19]. 

This mini-review describes the potential therapeutic applications of thermo-responsive P407 based-hydrogels containing various biocompatible nanocarriers as innovative formulations able to modulate the pharmacological and biopharmaceutical features of the drugs.

## 2. P407-Based Hydrogels

**Figure 1:**
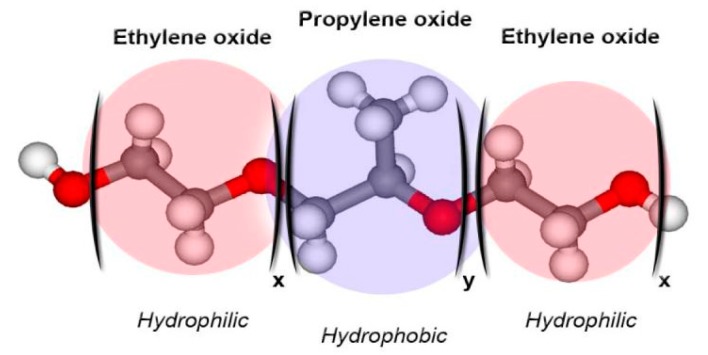
Schematic representation of the structure of poloxamer derivatives [22].

Poloxamer derivatives are a family of copolymers, characterized by different molecular weights of building blocks and the ratio between PPO and PEO units (Figure 1) [20]. Poloxamers are GRAS (generally recognized as safe) excipients widely used in the pharmaceutical industry [16,21,22].

P407, also known by the tradename Pluronic^®^ F-127 and Synperonic^®^ PE/F127, is an amphiphilic non-ionic surfactant of the triblock-copolymer family of poloxamers.

It is an excipient approved by the U.S. Food and Drug Administration (FDA) for pharmaceutical applications [18]. It has a molecular weight of about 12.6 kDa (PEO_101_PPO_56_PEO_101_) and is one of the most utilized thanks to its very low toxicity and weak immunogenic properties [18,23,24]. The non-toxic properties and stability of P407 aqueous “solutions” make them suitable as injectable formulations [25]. Moreover, the thermo-reversible properties of this material make them very interesting as drug delivery systems [18]. 

P407 molecules exist as unimers, surrounded by a hydration layer at low temperatures; when the temperature increases, a breakage of the hydrogen bonds between the aqueous solvent and the hydrophilic chains of the copolymer occurs, favoring hydrophobic interaction among the PPO blocks and the formation of micelles. The micellization depends on the concentration of the polymer because micelles are obtained when the amount of poloxamer is higher than the critical micelle concentration (CMC) [26]. A progressive increase in temperature induces the rearrangement of the P407-based micelles into a cubic structure and then into a hexagonal configuration promoting the gelation process [27]. The temperature at which the sol–gel transition occurs is defined as the low critical solution temperature (LCST) [28]. 

Solutions of P407 at copolymer concentrations of 15–30% (w/w) have an LCST similar to that of normal body temperature so they can easily be injected with a needle and syringe due to the initial low viscosity and will then promote the controlled release of an encapsulated drug by means of the gelation process [29,30]. 

Many experimental investigations have shown the potential applications of P407 gels as local drug delivery systems. Veyries and coworkers developed a P407-based hydrogel containing vancomycin, a broad-spectrum glycopeptide antibiotic used for the treatment of methicillin-resistant *Staphylococcus aureus* and other Gram-positive beta-lactam-resistant bacteria [25,31]. The drug is normally administered as a parenteral infusion, but its clinical application in the treatment of infections of poorly vascularized body compartments is limited due to its restricted tissue localization and the risks of toxicity associated with high serum concentrations [25,32,33]. Veyries and collaborators demonstrated a slow in vitro release of vancomycin from P407 hydrogels and a good tolerability in rats after a subcutaneous administration of the formulation. Moreover, a single dose resulted in a high local concentration of the drug for up to 24 h and provided significant antibacterial activity for as much as 8 days. This investigation clearly demonstrated that the antimicrobial activity of vancomycin was not altered by its inclusion in a matrix of P407, suggesting the potential role of this biomaterial in the development of formulations characterized by a controlled drug leakage [25]. 

P407-based hydrogels have also been successfully used in the treatment of pain [29,30,34,35,36,37]. Dos Santos and coworkers investigated the potentialities of P407-based hydrogels for the controlled release of tramadol (TR) by subcutaneous administration [34]. TR is a synthetic opioid analgesic usually employed in the treatment of pain of moderate intensity [38]. TR has also been proposed for the treatment of acute and chronic pain thanks to its atypical mechanism of action with respect to the classical opioids, which involves the modulation of the monoaminergic system by inhibiting noradrenaline (NA) and serotonin (5HT) reuptake at presynaptic terminals [34,39,40]. Moreover, the drug is characterized by a lower incidence of side effects even though the duration of the analgesic effects is relatively brief [41]. The entrapment of TR within P407 hydrogels promoted an increase in the duration of the analgesic effects as a consequence of a controlled and prolonged release of the drug for up to 48–72 h after subcutaneous injection while decreasing the cytotoxicity and the genotoxic effects as compared to the free drug [34]. 

**Figure 2:**
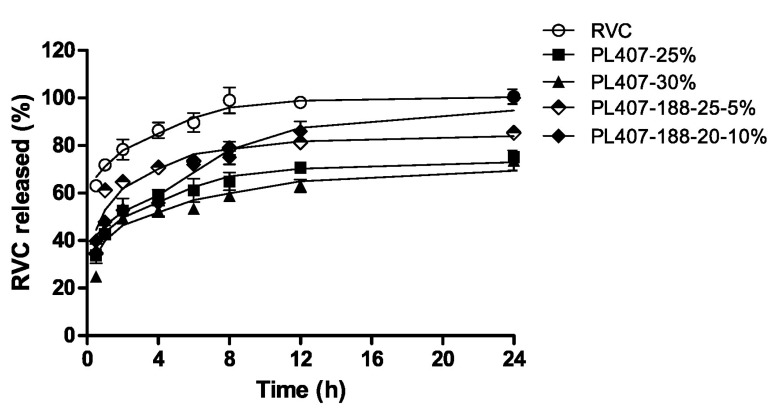
Release profiles of ropivacaine from poloxamer 407 (P407) and P407/poloxamer 188 (P188) hydrogels [42].

Akkari and coworkers used ropivacaine (RVC) as a model drug in order to evaluate the influence of its encapsulation within P407/poloxamer 188 (P188)-thermo-sensitive hydrogels for the treatment of post-operative pain following local administration [42]. In fact, P407 is often used in association with other poloxamers, especially with P188, in order to modulate the sol–gel transition temperature (T_sol–gel_) [16]. RVC is a long-acting local anesthetic widely used in surgical procedures and has turned out to be an interesting alternative to epidural administration, and used as an infiltrative anesthesia and as a means of post-operative pain relief [43]. Observation during in vitro release experiments revealed a slower RVC leakage from the hydrogel made up of the two copolymers with respect to the formulation prepared using P407 as a single agent or a solution of the drug (Figure 2). 

The entrapment of RVC in P407/P188 hydrogels increased the analgesic effects of the drug with no cytotoxicity or in vivo inflammatory reactions following local injection [42].

The previous studies and those described in the following paragraphs are just a few examples of the potential applications of these systems in the treatment of pain. Considering the peculiar nature of chronic pain, the strategies involved in its treatment are different and manifold. Although many therapeutic options are available, none of them is universally effective, and significant variation in the pharmacological effects has been observed as a consequence of the specific pathological condition of the treated patients [44]. 

Similarly, P407-based hydrogels have been used as delivery systems for a wide range of compounds including proteins and anticancer, anti-diuretic, and antiseptic drugs [24,27]. 

## 3. Biocompatible Nanocarrier-Loaded Poloxamer 407 Hydrogels

**Figure 3:**
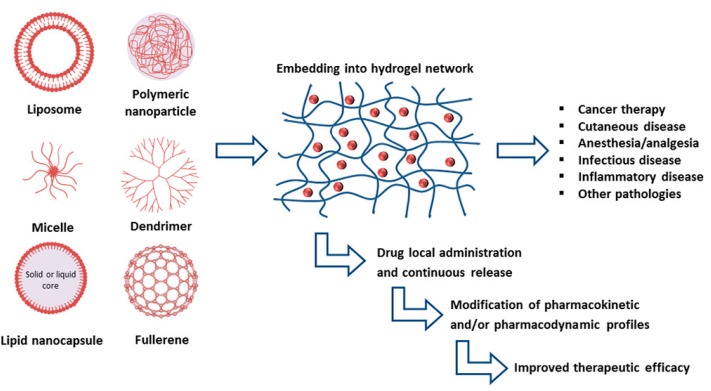
Advancements realized by the inclusion of various nanocarriers within hydrogels [4].

Despite their multiple benefits, P407-based hydrogels are characterized by a short residence time, due to their rapid dissolution in aqueous media and biological fluids [24]. Various approaches have been explored to overcome these limitations by adding bioadhesive polymers such as carbopol, sodium alginate, polycarbophil, hyaluronic acid, and chitosan to the structure with the aim of improving the strength, bioadhesiveness, and the residence time of the gel [45,46,47,48]. In addition, it has proved difficult to effectively encapsulate poorly water-soluble drugs in P407 hydrogels [49,50]. The inclusion of advanced drug delivery systems such as liposomes or polymeric nanoparticles in the hydrogel structure has offered interesting perspectives [18]. In recent years, the association of different pharmaceutical technologies has favored the development of innovative formulations able to improve the therapeutic efficacy of various drugs. In particular, nanocarrier-loaded gels have shown promising preclinical results for medical applications and for the treatment of a large number of diseases (Figure 3) [4]. 

Colloidal drug delivery systems are characterized by submicron particle sizes (typically less than 500 nm) and are able to deliver therapeutic agents characterized by different physico-chemical natures (hydrophilic, lipophilic, and amphiphilic) [51,52,53]. They can be divided into four classifications as a function of the material used: (phospho)lipid- (such as liposomes, solid lipid nanoparticles, nanostructured lipid carriers), polymeric- (for instance nanoparticles, nanoaggregates, dendrimers, protein-based systems), polysaccharide- (for example, cyclodextrins) and inorganic carriers. [54,55]. These systems are able to modulate the biopharmaceutical characteristics of the encapsulated drugs, improving their pharmacokinetic and biodistribution profiles, thereby minimizing toxicity and side effects [56]. Moreover, the entrapment of drugs into nanocarriers can increase their solubility and in vivo stability (e.g., peptides and oligonucleotides), prolong their plasmatic half-lives, and favor a controlled release and site-specific targeting [57,58,59,60]. Their inclusion in a gel can promote a better tissue residence time and a controlled release of the encapsulated compounds [4]. 

This strategy was used to increase the analgesic effect of bupivacaine, a short-acting local anesthetic, which was initially entrapped in a multivescicular system (Bupisome, DepoFoam^®^, Exparel^®^, Pacira Pharmaceuticals, Inc., Parsippany-Troy Hills, NJ, USA) that was successively included in a Ca-alginate cross-linked hydrogel (Bupigel^TM^) [61,62]. In vivo investigations demonstrated that a single administration of Bupigel^TM^ as a depot formulation in mice exerted a significantly prolonged analgesia when compared with the free solution of bupivacaine and Bupisome^TM^. In fact, the average duration of analgesia after a single administration of bupivacaine solution (1 h) increased up to 24 and 35 h when the drug was injected as Bupisome^TM^ or Bupigel^TM^, respectively [61].

Benzocaine (BZC) is another local anesthetic involved in the treatment of a variety of pain-related conditions as topical, dermal, and mucous formulations [63,64]. It is characterized by a rapid but brief pharmacological effect and its parenteral administration is restricted by its low water solubility and toxicity [65]. Mura and coworkers developed a BZC-loaded liposomal hydrogel made up of Carbopol^®^ 940. In vitro permeation studies through artificial membranes showed that a decrease in drug permeation came about as well as a slow release promoted by the polymeric network surrounding the vesicles with respect to the liposomal formulation [66]. In vivo studies performed on rabbits using the conjunctival reflex test demonstrated a significant increase in both the intensity and the duration of the anesthetic effect of BZC when formulated as a liposomal hydrogel with respect to the free drug form [66]. 

Nanocarrier-loaded gels have also been successfully used in order to modulate the biopharmaceutical properties of other active compounds, and several clinical trials are in progress [4,67,68,69]. In the following sections, the state of the art concerning the development of formulations made up of biodegradable nanosystems entrapped within P407 hydrogels is discussed (Table 1).

### 3.1. Vesicular Delivery Systems Embedded in P407 Hydrogels

Vesicular carriers are systems made up of biocompatible materials (usually phospholipids or non-ionic surfactants) able to arrange themselves in peculiar structures made up of bilayers containing aqueous compartments; they can retain drugs characterized by different physico-chemical properties favoring the encapsulation of (i) water-soluble compounds in aqueous compartments; (ii) lipophilic derivatives within the lamellar membrane; and (iii) amphiphilic molecules at the interface [83,84]. The particular physico-chemical properties and the characteristics of extreme biomimetism and biodegradation make these systems versatile and suitable carriers of various bioactives such as proteins, vitamins, enzymes, polypeptides, genetic material, antibodies, and synthetic drugs [85].

#### 3.1.1. Liposomes

Liposomes were the first vesicular carriers developed. They are self-assembled vesicles having the same supramolecular lipid organization as the natural membranes of cells and various liposomal formulations (i.e., Doxil^®^, Caelyx^®^, Myocet^®^, AmBisome^®^, DepoDur^TM^, DepoCyt^®^, etc.) are presently on the market and are being used in clinical practice [86,87]. 

The experimental inclusion of liposomes in hydrogels promoted a sustained release of the drug entrapped in the vesicular structure as a consequence of the increased residence time at the administration site [88,89]. Hydrogels can stabilize liposomes due to the modulation of their rheological properties, favoring the topical administration of vesicles [90,91]. The release of the entrapped compounds depends on different factors related to the physico-chemical properties of the drug and to the stability of the liposomes dispersed in the semisolid formulation [89,91]. 

**Figure 4:**
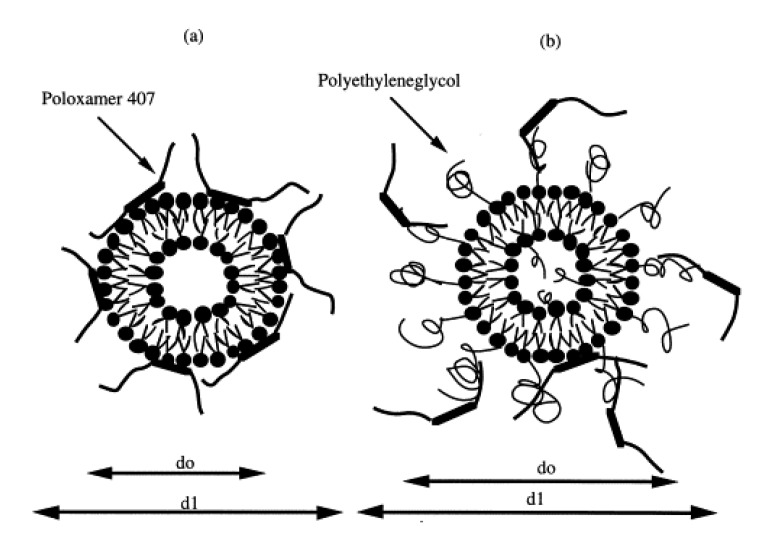
Mechanism of interaction between P407 and conventional (**a**) or sterically stabilized (**b**) liposomes. (d_0_ = diameter of liposomes); (d_1_ = diameter of liposomes embedded in P407) [92].

Bochot and coworkers investigated interaction between P407 hydrogels and various liposomes characterized by different lipid compositions and surface charges [92]. Adsorption studies showed that P407 strongly interacted with both negatively and positively charged liposomes, even though the best interaction was obtained using cationic or neutral non-sterically stabilized vesicles. Moreover, the encapsulation of a fluorescent hydrophilic probe showed a sustained release of the compound from the conventional liposomes (made up of phospholipids and cholesterol) when they were embedded in P407 hydrogels, while a slow leakage of the molecule was observed when a polyethylene glycol (PEG) derivative was added to the liposomal composition. This phenomenon was explained hypothesizing an intrusion of P407 residues within the phospholipid layers able to promote the formation of pores, fractures, or regions of high-fluidity; contrarily, the use of a PEG-derivative provided a steric barrier on the vesicular surface that decreased its interaction with the copolymer (Figure 4). In addition, the rheological analyses demonstrated that the thermo-sensitive properties of P407 were not affected by the inclusion of liposomes in the hydrogel, confirming the rationale of using the obtained formulation for topical administration [92].

The same research team investigated the delivery of the oligonucleotide pdT16 by liposomes entrapped in P407 hydrogel with the aim of obtaining a controlled ocular release of the compound [70]. It has been shown that antisense oligonucleotides have a significant inhibitory effect on DNA viruses responsible for ocular diseases, such as herpes simplex virus and human cytomegalovirus, but their use is limited by their poor stability in biological fluids [93,94]. The encapsulation of genetic material within nanocarriers can protect it from degradation as well as prolong its pharmacological activity [95,96]. In addition, the inclusion of oligonucleotide-loaded nanocarriers in a thermo-sensitive hydrogel favors a prolonged retention of the vesicular formulation at the site of administration [97]. The liposomes used to entrap pdT16 were not destabilized by the P407 gel (27% *w*/*v* of copolymer) and the release of the oligonucleotide decreased significantly as compared to a P407 hydrogel containing genetic material in free form, confirming the “reservoir” effect exerted by the mixed system [70].

These formulations have also been employed for other administration routes. Mura and coworkers proposed a P407 hydrogel containing liposomes for the nasal delivery of opiorphin (OPI) [71]. OPI is a natural peptide found in human saliva that appears to be an interesting and promising therapeutic agent in the treatment of acute and chronic pain, thanks to its strong analgesic effect similar to that of morphine but without its side effects. Unfortunately, its clinical application is limited by its short half-life following intravenous administration due to its rapid degradation by the peptidases present in the bloodstream [98,99]. The encapsulation of OPI in PEGylated liposomes dispersed in a P407 hydrogel promoted the nasal residence time of the peptide and modulated its release without any influence on the rheological features of the system. Moreover, ex vivo permeation studies, carried out on excised porcine nasal mucosa, evidenced that the liposomal hydrogel formulation provided a sustained and controlled delivery of opiorphin, confirming the role of the nanocarriers in the increased permeation of the compound through the nasal mucosa as compared to gel containing the peptide in the free form [71]. 

Bleomycin A6 (BLM A6) is a glycopeptide produced by the bacterium *Streptomyces verticillus,* characterized by antibiotic and antitumoral activity [72]. It acts by means of DNA cleavage through the production of reactive oxygen species following complexation with Fe^2+^ ions [100]. However, high and frequent doses of BLM A6 are required due to its short half-life and fast clearance [101]. The goal of Ding and coworkers was to develop an anionic liposomal formulation containing BLM A6 that was dispersed in a thermo-sensitive P407 hydrogel in order to prolong the release rate of the drug and its pharmacological efficacy [72]. The negative charge (due to the phosphatidylglycerol) of the surfaces of the vesicles enhanced viscosity and gel strength at 37 °C, avoiding the rapid corrosion of the polymeric matrix under physiological conditions and in vivo fluorescence imaging performed on Kumming mice demonstrated that this formulation remained at the administration site for at least five days [72].

Paclitaxel (PTX) is another well-known antineoplastic agent, which acts by means of different mechanisms, involving mitotic inhibition by the hyperproliferation of microtubules. It is widely used in the treatment of a wide variety of cancers, such as ovarian, skin, breast, pancreatic, and lung cancers [73,102]. However, several side effects have been reported when PTX is administered intravenously as a consequence of the necessity of using organic solvents in its preparation due to its poor aqueous solubility. To reduce these risks, patients are routinely pretreated with corticosteroids and antihistamines, and colloidal formulations have been developed (Abraxane^®^: Celgene Europe BV, Utrecht, Netherlands; Lipusu^®^: Nanjing Luye Sike Pharmaceutical Co. Ltd., Nanjing, China) in order to avoid the use of organic solvents [103,104]. Nie and coworkers described an in situ gel system made up of liposomes containing PTX dispersed within a P407 hydrogel with the aim of modulating the release kinetic of the active compound and increasing its antitumor efficiency [73]. The in vitro release experiments showed a prolonged leakage of PTX from this formulation that was significantly greater as compared to those obtained using a gel formulation containing the drug in free form or the commercial formulation of the bioactive (Taxol^®^: Bristol-Myers Squibb Co., Princeton, NJ, USA). In addition, it was demonstrated that the increase in the lipid content in the gel opposed the undesirable dissolution of the gel. This phenomenon was taken advantage of to avoid the toxicity exerted by the high concentrations of poloxamer needed to modulate the rheological features of the gel. The inclusion of liposomal PTX in the P407 hydrogel improved the stability of the colloidal formulation; namely, a destabilization of PTX-loaded liposomes occurred after 7 days at room temperature evidenced by a certain sedimentation and aggregation, while in the case of liposomal gel formulations none of the previously described phenomena was observed for up to 30 days [73]. Finally, the evaluation of the in vitro cytotoxicity on human oral KB cancer cells demonstrated a better anticancer activity of PTX after its encapsulation in liposomes dispersed in a poloxamer matrix with respect to poloxamer-free liposomal systems, evidencing the potential role of the copolymer as an intracellular penetration enhancer [73]. 

A similar approach for delivering PTX was recently proposed by Mao and coworkers who developed a mixed P407/P188 (24:1, *w*/*v*%) hydrogel containing a liposomal formulation of the drug [74]. This formulation inhibited the tumor growth of a xenograft tumor mouse model of ascites sarcoma for up to 12 days following a single subcutaneous injection. Moreover, no weight loss of tumor-bearing mice was noted and only a slight toxicity in healthy tissues, especially the heart and lungs, was observed after the intratumor injection of the formulation [74].

#### 3.1.2. Niosomes

Niosomes are nonionic surfactant vesicles similar to liposomes, obtained by the self-assembly of nonionic amphiphiles in aqueous media [1,105,106]. The low cost, the great physical stability, and the availability of surfactants led to the use of these colloidal carriers as an alternative to conventional liposomes [106,107]. The influence of niosomes on the rheological properties of P407 was investigated by Antunes and coworkers [75]. In detail, they prepared P407 hydrogels containing Tween-60-based niosomes and investigated the rheological behavior of the obtained formulation and the permeation profile through rabbit ear skin using a suitable Franz-cell apparatus. The niosomes did not modify the gelation properties of P407 but rather increased the gel viscosity as a consequence of the more rigid network obtained by the strong interactions that come about between the copolymer and the vesicles. This fact influenced the leakage of the model drug sodium diclofenac from the niosomes especially when the high concentrations of P407 needed to diffuse through the various barriers (i.e., colloidal bilayer and polymeric network) were used [75]. 

A similar approach was employed by Fathalla and coworkers, who prepared and characterized Span-based niosomes embedded in P407 hydrogel in order to obtain the sustained release of aceclofenac, a widely used anti-inflammatory agent used in the treatment of rheumatoid arthritis [108].

#### 3.1.3. Ethosomes

In 1997, Touitou et al. developed innovative soft vesicular carriers made up of phospholipids and ethanol, named ethosomes [109,110,111]. Unlike liposomes, ethosomes penetrate the skin better thanks to an enhanced bilayer fluidity, malleability, and deformability [112,113,114]. Shelke and coworkers encapsulated zolmitriptan (ZMT) in ethosomes and dispersed the vesicles in a mucoadhesive hydrogel made up of P407, Carbopol 934, and hydroxypropyl methylcellulose K 100, with the aim of increasing the brain localization of the drug following nasal administration [76]. ZMT is the drug of choice for the treatment of migraine headaches, but its low oral bioavailability compromises its pharmacological efficacy and the multiple administrations that are necessary induce several side effects [115]. In the last decade, nasal drug delivery is emerging as a potential approach to be used for the topical administration of drugs to reach the brain, thanks to the existence of a direct connection between the olfactory region of the nasal cavity and the cerebral-spinal fluid [116]. In vitro and ex vivo permeation studies performed on cellophane dialysis membrane and sheep nasal mucosa membrane demonstrated that the entrapment of ZMT in ethosomes embedded in a mucoadhesive P407-based hydrogel dramatically increased the permeability coefficient of the drug, modulated the release rate of the bioactive, and showed no toxicity of the mucosal tissue, confirming the potential application of this formulation for the treatment of migraines by a non-invasive route [76].

### 3.2. Solid Lipid Nanoparticles (SLNs)

SLNs are another class of colloidal drug delivery systems composed of biocompatible and biodegradable lipids (i.e. triglycerides, partial glycerides, fatty acids, steroids, and waxes) [117]. They are characterized by a solid lipophilic matrix able to retain lipophilic compounds, enhancing their physical stability and providing protection against chemical degradation [83,118]. 

Mirza et al. developed a formulation made up of SLNs containing itraconazole dispersed in a P407-based gel with the aim of increasing the therapeutic efficacy of the drug [77]. Itraconazole is a potent antifungal compound used in the treatment of vaginal candidiasis, usually administered in the form of oral formulations that are characterized by scarce bioavailability [119]. The hydrogel containing the active compound encapsulated within SLNs exhibited the desired gelling temperature and viscosity as well as a suitable degree of drug leakage (62.2% of entrapped drug was released over a 20-hour period); moreover, in vivo investigations demonstrated that no irritation was provoked by the formulation following topical application in Wistar rats, while the antimicrobial effect of the drug was significantly greater than the marketed formulations [77].

Thermo-sensitive P407-based hydrogels are widely used for the development of “liquid suppositories” that can be easily administered in liquid form becoming semi-solid in situ and hence more acceptable to patients with respect to the conventional rectal dosage forms [120]. It has been demonstrated that P407 hydrogel can increase the bioavailability of nimesulide, ketoprofen, diclofenac, and other anti-inflammatory drugs administered rectally [121,122,123]. Din et al. developed a novel SLN formulation containing flurbiprofen embedded in a P407 hydrogel for the rectal administration of the drug in order to improve its bioavailability [78]. Flurbiprofen, in fact, is an anti-inflammatory compound characterized by a low degree of bioavailability due to its poor water-solubility, and it irritates gastric tissue following oral administration. It also has several side effects such as physical and mental discomfort and atrophy or hypertrophy of subcutaneous fat is described when the bioactive is injected parenterally [78,124]. A lipid mixture of tricaprin and triethanolamine was used to obtain SLNs characterized by a mean diameter of ~190 nm able to retain flurbiprofen; the nanosystems were embedded in P407/P188 hydrogels modulating the concentrations of the various components in order to obtain a formulation suitable for rectal administration. The system made up of 10% *w*/*w* SLNs, 15% *w*/*w* P407, and 25% *w*/*w* P188 assumed a liquid physical state at room temperature (i.e., easy to administer rectally), while it gelled at 35 °C (inside the body). The pharmacokinetic investigations showed an increased half-life of the drug encapsulated in the SLN/P407 system as compared to both the formulation obtained by the direct dispersion of the flurbiprofen within the hydrogel and the free form of the active compound; moreover, a lower initial plasmatic concentration was observed as a consequence of the slow release of the drug promoted by SLNs contained in the P407 matrix. There was no toxicity of the rectal mucosa [78].

### 3.3. Polymeric Micro- and Nanoparticles Embedded in P407 Hydrogels

Polymeric particles are drug delivery systems able to entrap various active compounds as a function of the polymer of which they are composed. Generally, they can be classified as micro- and nanoparticles when their mean diameter is greater than or less than 1000 nm, respectively [53,125]. Many biodegradable or biocompatible polymers have already been approved by the U.S. FDA and the EMA for pharmaceutical application and many of them have been used to develop innovative micro- and nanoparticles [60,126]. Among these, poly(D,L-lactide) (PLA), poly(D, L-glycolide) (PLG), and their copolymers poly(lactide-co-glycolide) (PLGA), polyalkylcyano acrylate (PACA), poly-ε-caprolactone-derivatives, chitosan, alginate, gelatin, and albumin are polymers extensively used in preformulation and preclinical investigations [127,128]. 

Polymeric nanoparticles (NPs) have been extensively evaluated as drug nanocarriers with the aim of improving the therapeutic efficacy of water soluble/insoluble bioactive compounds, improving their bioavailability and solubility and increasing their localization in specific tissues [129,130,131]. These systems are classified as spheres (a matrix containing dispersed or adsorbed drugs) and capsules (characterized by a water/oil core surrounded by a polymeric shell); they have been used as drug delivery systems for the treatment of various diseases, such as cancer, AIDS, diabetes, malaria, and tuberculosis [126,129]. 

Shen and coworkers encapsulated PTX in PEG-PLGA–conjugated to poly(L-lysine)-cyclic peptide (arginine-glycine-aspartic- glutamic-valine acid) NPs [79]. The nanosystems were successively dispersed in a P407-based thermo-sensitive hydrogel, and the antitumor efficacy of this formulation was tested on orthotopic and xenograft pancreatic-tumor mouse models through direct intratumor and subacutaneous injection. The PTX administered as a nanoparticle/P407hydrogel formulation was retained in the tumor mass for up to 50 days as compared to both the PTX NPs and the free drug form (23 and 3 days, respectively), permitting a gradual release of the bioactive compound and a constant therapeutic effect [79]. 

In another experimental work, Timur and coworkers combined the properties of chitosan NPs containing tenofovir (TFV), an antiviral agent used in the treatment of the human immunodeficiency virus (HIV), together with the characteristics of P407 in order to develop a muco-adhesive and thermo-gelling system for the vaginal delivery of the drug [80]. A certain amount of free TFV was also encapsulated in the gel in addition to chitosan NPs, with the aim of decreasing the release rate of the bioactive compound from the colloidal systems. In fact, in vitro investigations evidenced two peculiar drug release features of this formulation: (1) a burst-release effect, induced by the presence of the free drug within the gel network, and (2) a sustained drug release for up to 24 h deriving from its leakage from the chitosan NPs [80].

Kaur et al. developed an in situ muco-adhesive gel (P407/HPMC) containing tramadol-loaded chitosan nanoparticles to treat depression by means of nasal applications [81]. Depression is a multifactorial disease and one of the most familiar mental disorders in humans, caused by a deficiency of norepinephrine and serotonin [132]. The main limitation of the drugs used for the treatment of this disease is related to the difficulty of their reaching the brain from the circulatory system. The nasal administration of tramadol-loaded NPs dispersed within a hydrogel significantly attenuated the induced depressive status in Wistar rats with respect to other formulations of the drug, confirming once again the potential of intranasal administration as an effective route for reaching the brain [81]. 

Vaccines are the most cost-effective life-saving medications in history, protecting populations from many infectious diseases [133]. Despite recent progress, improvements are still required due to concerns about weak immunogenicity, intrinsic instability, and toxicity in vivo, besides the need for multiple administrations. To overcome these limitations, nanotechnology platforms have been recently introduced in vaccine development for the prevention and treatment of infectious diseases [134]. Plasmid DNA (pDNA) vaccines have been shown to act against a wide range of diseases but have low efficacy and require multiple administrations due to their poor cell uptake and rapid degradation by the nucleases [135]. Bansal et al. developed a vaccine formulation for the treatment of rabies made up of pDNA-loaded PLGA/chitosan nanoparticles embedded in P407 hydrogel [82]. Stability analyses revealed that the pDNA contained in the PLGA-chitosan nanosystems was stable after incubation at high temperatures and the inclusion of the colloids in the hydrogel enhanced the retention time of the drug at the administration site, besides bettering its cellular uptake by resident muscle cells and antigen-presenting cells; the formulation also prevented its distribution to other body compartments [82]. 

In a recent investigation, Khan et al. exploited the inclusion of polymeric microparticles in P407/chitosan hydrogel to enhance the ocular bioavailability of tobramycin sulfate [136]; in fact, microparticles usually show slower elimination kinetics from the precorneal compartment with respect to nanoparticles even though their sizes must be less than 10 µm to be tolerated [137]. The described formulation promoted a sustained release together with a significant ocular permeation of tobramycin sulfate along with a greater drug concentration in the aqueous humor of albino rabbits as compared to the commercial formulation of the drug (Toba eye drops: Sun Pharma Industries Ltd., Mumbai, India). There was also no evidence of ocular irritation [136]. 

Microparticles dispersed in thermo-sensitive P407 hydrogels were also used for the treatment of pain. Chen et al. developed an injectable formulation made up PLGA microparticles-embedded in P407 hydrogel with the aim of prolonging the pharmacological effect of lidocaine [138]. In vivo testing, performed by monitoring the sensory and motor functions of rats following sciatic nerve block and the use of a hot-plate, showed that the described formulation achieved the maximal nerve block within 30 min, suggesting that lidocaine-loaded microspheres could immediately relieve the pain of patients after surgery. Moreover, the drug-loaded microspheres enclosed in P407 gel yielded the longest duration of sensory and motor block (up to approximately 8.5 h), with respect to the effect exerted by the P407-free microparticles containing lidocaine (5 h), a gel formulation of the drug (5 h), and a saline solution of the bioactive (2 h) [138]. 

Recently, Hu and coworkers combined the advantages of micro- and nano-encapsulation to obtain the co-delivery of various compounds [139]. Namely, they encapsulated PTX-nanoparticles and lapatinib (LAPA) microparticles in a thermo-sensitive P407 hydrogel in order to obtain a local, injectable, peritumoral delivery system. LAPA is a potent and selective human epidermal growth-factor-receptor-2 tyrosine kinase inhibitor, but its clinical use is compromised by its extensive albumin binding, poor oral bioavailability, and poor aqueous solubility [140,141].

In fact, commercial LAPA (Tykerb^®^: Novartis Pharmaceuticals Corporation, East Hanover, NJ, USA) is usually administered orally, but since it only allows a limited amount of the drug to be delivered to the tumor site, it must be administered in large dosages, which causes side effects [139,142,143]. In vitro and in vivo analyses of hydrogels containing nanoPTX and LAPA-loaded microparticles showed an increased anticancer efficacy with respect to the drugs tested as single formulations (free forms or encapsulated in the nano/micro-systems) following peritumoral injection; moreover, a lower degree of toxicity resulted, as compared to that for the oral formulation of LAPA [139].

## 4. Conclusions

P407-based hydrogels are attractive pharmaceutical formulations for medical application. The peculiar thermo-reversible behavior they exhibit makes P407-based hydrogels suitable and versatile pharmaceutical formulations. They are easy to manage during the manufacturing process and administration, thanks to their liquid state at room temperature. The subsequent sol–gel transition establishes a prolonged and sustained drug release of entrapped compounds, decreasing their efficacious dosage, side effects, and the number of required administrations. However, the weak mechanical properties of P407-based hydrogels, their rapid dissolution when they interact with physiological media, and their short residence time are critical points that need to be properly evaluated and modulated for their medical application [24,144,145]. Edsman et al. evaluated the ocular residence time of P407-based hydrogels (18–25% w/w of copolymer) containing fluorescent microparticles in human subjects. They observed that a rapid elimination of the fluorescent microparticles takes place, because the tear fluid of the eyes dilutes and washes them away [146]. P407 exhibits scarce bioadhesive properties probably because of its weak mechanical strength [24,147,148]. Drug release also depends on the strength of the hydrogel, which is proportional to the concentration of copolymer employed [149,150]. Unfortunately, an increase in its concentration causes a reduction in the T_sol–gel_ rendering the system semi-solid at room temperature and compromising administration [48,151].

The association of P407-based hydrogels and nanocarriers is a promising strategy for obtaining a sustained drug release without using high concentrations of polymer.

Moreover, nanocarriers offer important advantages as drug delivery systems, such as site-specific targeting, prolonged systemic residence time and protection of the entrapped compounds from metabolic pathways. In this mini-review, we have described several examples of the advantages deriving from the association of different kinds of nanocarriers and P407-based hydrogels. However, to the best of our knowledge, only one formulation made up of P407 has been approved by the U.S. FDA. for temporary endovascular occlusion of blood vessels up to 4 mm in diameter below the neck (LeGoo^®^, Pluromed Inc., Woburn, MA, USA). The numerous experimental investigations going on in the absence of human clinical trials makes it necessary to further evaluate the toxicity and potential side effects induced by P407-based hydrogels and the real benefit-to-risk ratio of using these formulations as innovative medicines. Indeed, the lipid profile alteration, the possible renal toxicity, and the immunomodulation provoked by P407 after parenteral administration are possible limitations for the use of this material in clinical practice [18]. 

## Figures and Tables

**Table 1:** Examples of drug-loaded biocompatible nanocarriers embedded in P407-based hydrogels.

Type of Nanocarrier	Formulation	Composition of Nanocarrier	Gel Composition	Reference
**Vesicular Systems**				
Liposomes	pdT16-loaded liposomes dispersed in a P407-based hydrogel as innovative ocular controlled release formulation.	SPC ^1^, CHOL ^2^, PEG-DSPE ^3^	P407 ^4^	[70]
	Liposomal mucoadhesive thermo-sensitive gel for the nasal delivery of opiorphin (OPI).	EPC ^5^, CHOL, SA ^6^, PEG-DSPE	P407, Carbopol 934P	[71]
	Bleomycin A6 (BLM A6)-loaded liposomes dispersed within thermo-sensitive in-situ gel for a sustained drug release.	PC S100 ^7^, DPPG ^8^, CHOL	P407, P188 ^9^	[72]
	Paclitaxel (PTX)-loaded liposomes embedded in P407 gel for a controlled drug release and an improved antitumor efficiency.	SPC	P407	[73]
	Thermo-sensitive hydrogel system containing PTX-liposomes for the local treatment of tumors.	SPC, CHOL	P407, P188	[74]
Niosomes	Tween 60-based vesicles embedded in P407 hydrogel as a transdermal drug delivery system.	Tween 60 ^10^	P407	[75]
Ethosomes	P407-based thermo-sensitive hydrogel containing zolmitriptan (ZMT)-loaded ethosomes for an efficacious brain targeting and a sustained drug release.	Soya lecithin, ethanol	P407, Carbopol 934, HPMC ^11^ K100	[76]
**Solid lipid nanoparticles (SLNs)**				
	SLNs dispersed in a P407-based gel as delivery system for itraconazole.	Stearic acid, Compritol 888, Compritol E ATO	P407, Carbopol 934	[77]
	SLNs-loaded dual-reverse thermo-sensitive hydrogel for rectal administration of flurbiprofen.	Tricaprin, triethanolamine	P407, P188	[78]
**Polymeric nanoparticles (NPs)**				
	Thermo-sensitive gel containing paclitaxel-loaded NPs as delivery system for pancreatic tumor therapy.	mPEG-PLGA-PLL-cRGD ^12^	P407, P188, HPMC, MC ^13^, Sodium Alginate	[79]
	Tenofovir–loaded NPs embedded in vaginal gels for the prevention of HIV infections.	Chitosan, TPP ^14^	P407	[80]
	Intranasal in-situ gel with nanoparticulated polymeric carriers for brain delivery of tramadol.	Chitosan, TPP	P407, HPMC K15M	[81]
	Rabies pDNA nanoparticulate vaccine in P407 hydrogel.	PLGA ^15^	P407	[82]

^1^ soybean phosphatidylcholine; ^2^ cholesterol; ^3^ 1,2-distearoyl-sn-glycero-3 phosphatidylethanolamine-N(poly(ethyleneglycol)-2000); ^4^ poloxamer 407; ^5^ egg phosphatidylcholine; ^6^ stearylamine; ^7^ phosphatidylcholine, ^8^ dipalmitoylphosphatidylglycerol; ^9^ poloxamer 188; ^10^ polyoxyethylene sorbitan monostearate; ^11^ hydroxy propyl methyl cellulose; ^12^ monomethoxy (polyethylene glycol)-poly(D,L-lactide-co-glycolide)-poly(L-lysine)-cyclicpeptide (arginine-glycine-asparticglutamic-valineacid); ^13^ methyl cellulose; ^14^ tripolyphosphate; ^15^ poly (D, L-lactide-co-glycolide).
